# Orthorhombic polymorph of 4-(2,2′:6′,2′′-terpyridin-4′-yl)aniline

**DOI:** 10.1107/S2414314626001069

**Published:** 2026-02-10

**Authors:** Nicola Edwards, Shao-Liang Zheng

**Affiliations:** a Department of Chemistry, University of St. Joseph, West Hartford, CT, 06032, USA; b Department of Chemistry and Chemical Biology Harvard University,Cambridge MA 02138 USA; Howard University, USA

**Keywords:** crystal structure, aniline, terpyridine

## Abstract

Crystallographic data for the title compound, C_21_H_16_N_4_, are reported herein. The compound was recrystallized from a methanol/aceto­nitrile solvent system at 298 K. It crystallizes in the *Pca*2_1_ space group at 100 K compared to the previously reported monoclinic polymorph and displays inter­molecular hydrogen bonding, through N—H⋯N contacts, and π–π inter­actions. None of the rings in the structure is coplanar – dihedral angles defined by atoms of the peripheral rings with the central pyridine range from −7.1 (3) to 29.8 (3)°.

## Abstract 2

Crystallographic data for the title compound, C_21_H_16_N_4_, are reported herein. The compound was recrystallized from a methanol/aceto­nitrile solvent system at 298 K. It crystallizes in the *Pca*2_1_ space group at 100 K and displays inter­molecular hydrogen bonding, through N—H⋯N contacts, and π–π inter­actions.

## Graphical abstract

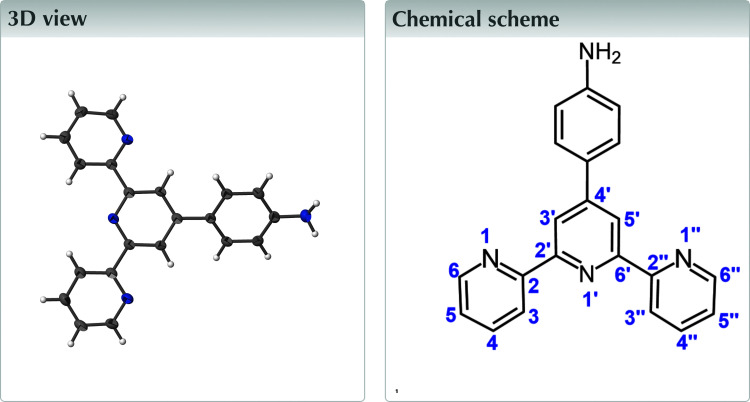

## Structure description

Amines, a class of compounds that possess at least one C—N bond, are indispensable in organic chemistry. They are utilized extensively as starting materials and reagents in the synthesis of compounds involving reductive amination, nucleophilic substitution and amide coupling reactions (Afanasyev *et al.*, 2019 ▸; Dunetz *et al.*, 2016 ▸; Mondal & Malakar, 2020 ▸). Amines are key moieties in biologically active compounds such as anti­histamines, anti­depressants and anti­psychotics and hence these moieties are featured in a vast number of studies in medicinal and pharmaceutical chemistry (George *et al.*, 2026 ▸; Qurrat-ul-ain *et al.*, 2024 ▸). Expansion of synthetic methodologies for amines has led to a plethora of new structures with a host of chemical and physical properties (Li *et al.*, 2016 ▸; Salvatore *et al.*, 2001 ▸; Umar & Luo, 2023 ▸; Afanasenko *et al.*, 2025 ▸). Amines are also used as building blocks in the synthesis of polymers, sensors and as catalysts (Tanaka, 2023 ▸; Froidevaux *et al.*, 2016 ▸). The properties of amines are due in part to their ability to engage in hydrogen bonding and other inter­molecular inter­actions, and hence there is keen inter­est in the structural characterization of amines.

The single-crystal X-ray analysis of the title compound, C_21_H_16_N_4_, in the space group *Pca*2_1_, is reported herein. This compound was recrystallized from a methanol/aceto­nitrile solvent system at 298 K. This moiety is known for its ability to coordinate metals and is featured in mol­ecules that serve as ligands, polymers and catalysts (Schubert *et al.* 2011*a* ▸,*b* ▸; Winter & Schubert, 2020 ▸; Kainat *et al.*, 2024 ▸).

**Figure 1:**
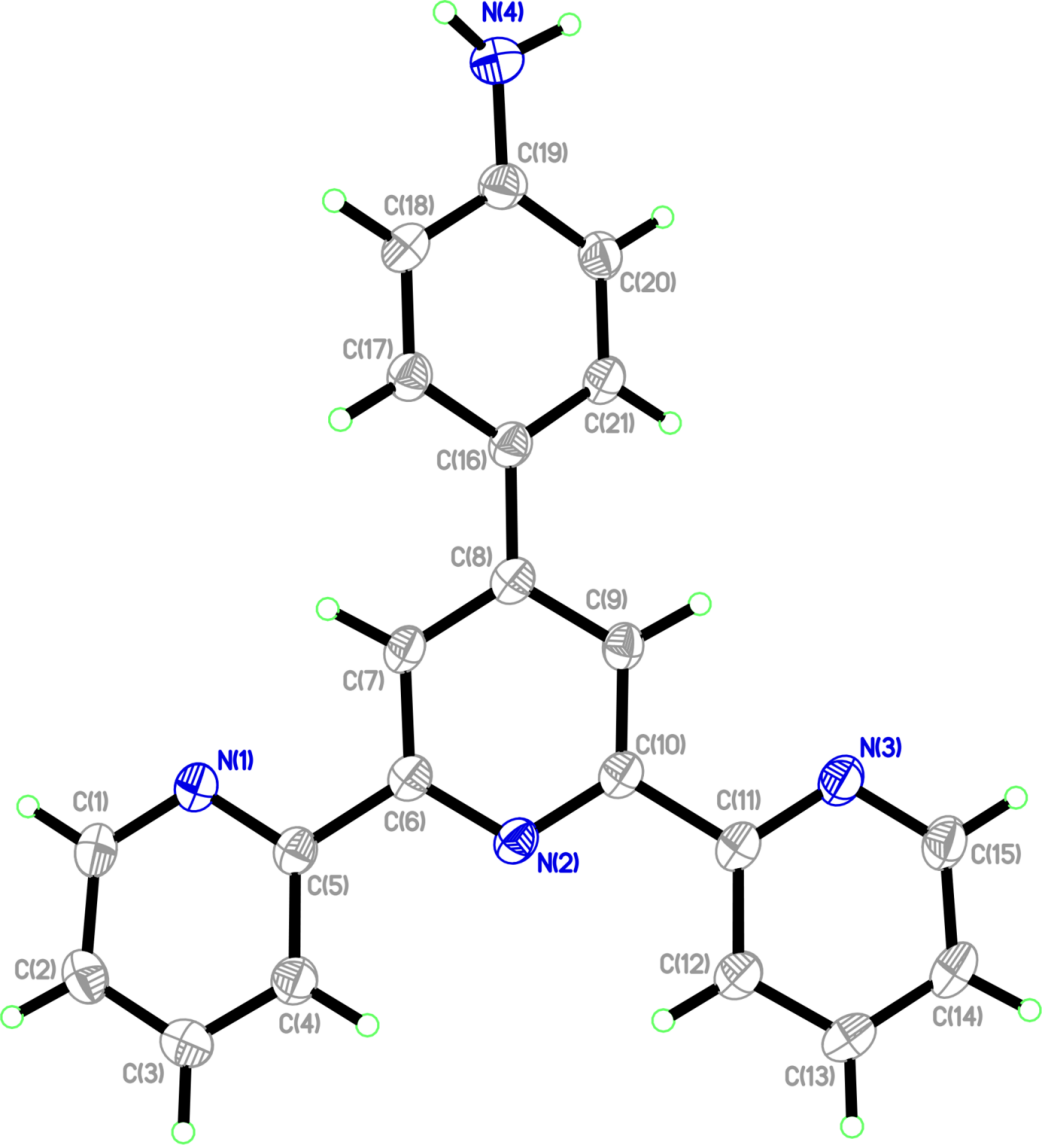
Perspective view of the title compound with the atom-numbering scheme showing 50% probability displacement ellipsoids.

The mol­ecular structure of the title compound is shown in Fig. 1 ▸. It features the 2,2′:6′,2′′ terpyridine moiety in the *trans trans* conformation with respect to the pyridyl nitro­gen atoms. The degree of coplanarity of the rings was determined by dihedral angles formed by atoms of the peripheral rings with atoms in the central pyridine ring. The dihedral angles formed by atoms C9—C10—C11—N3, N1—C5—C6—C7 and C9—C8—C16—C21 were determined to be −7.1 (3), 11.8 (3) and 29.8 (3)° respectively indicating that none of the rings is coplanar; the greatest deviation from planarity evidenced by benzene ring bearing the amino group and the central pyridine.

**Figure 2:**
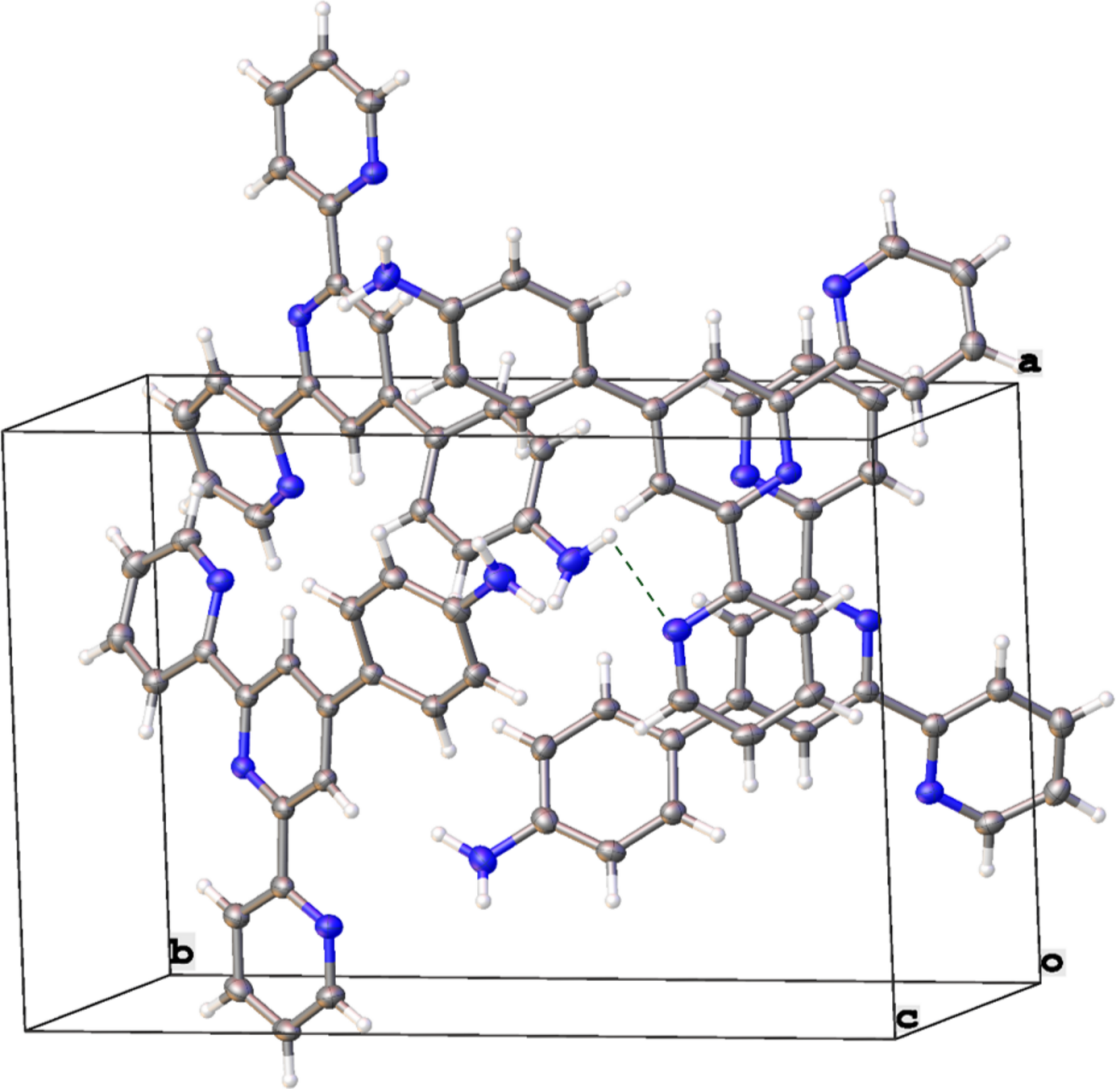
The unit cell showing the inter­molecular N—H⋯N and π–π inter­actions.

In the crystal, mol­ecules are linked by inter­molecular N—H⋯N inter­actions, forming a three-dimensional network (Table 1 ▸, Figs. 2 ▸ and 3 ▸). In addition, the structure includes π–π inter­actions (Figs. 2 ▸ and 3 ▸) with the distances between two pyridine ring planes being 3.6273 (19) Å [slippage = 1.451 (3) Å] and 3.417 (2) Å [slippage = 1.893 (3) Å].

**Figure 4:**
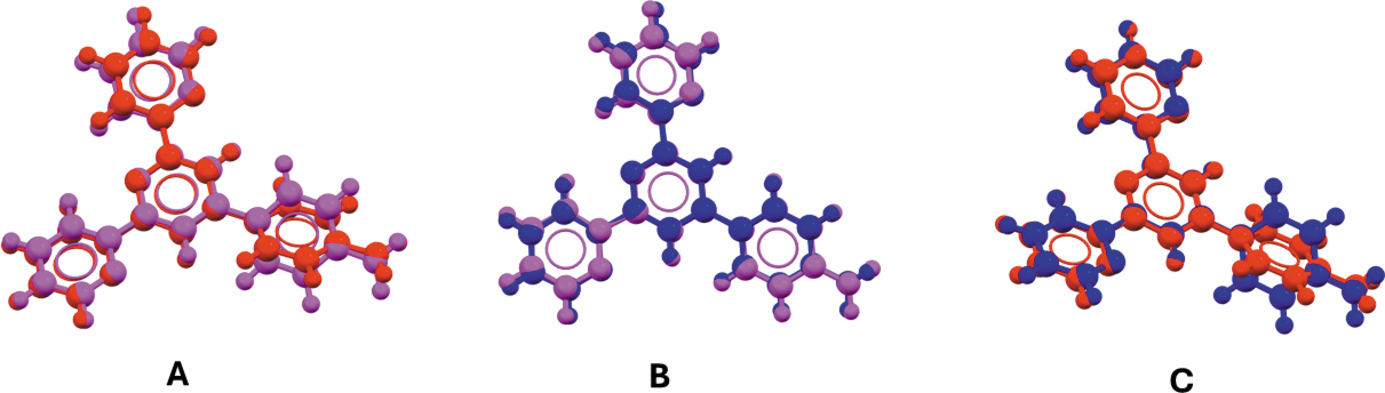
(A) The overlap of the two crystallographically independent mol­ecules of the title compound in the unit cell reported by Storrier *et al.* (1997 ▸) (B and C) The overlap of the crystallographically independent mol­ecule in the asymmetric unit in this data set and the two crystallographically independent mol­ecules of the title compound in the asymmetric unit reported by Storrier *et al.* (1997 ▸) calculated using *Mercury* (Macrae *et al.*, 2020 ▸).

Crystals of this compound were previously obtained from a chloro­form–methanol solution by Storrier and co-workers, and single-crystal X-ray data in the *P*2_1_/*c* space group were reported (Storrier *et al.*, 1997 ▸). Two crystallographically independent mol­ecules were found in the asymmetric unit of the previous data set. These mol­ecules differ primarily in the orientation of one pyridine ring (Fig. 4 ▸*a*), with a maximum distance between equivalent atoms (Max·D), calculated using *Mercury* (Macrae *et al.*, 2020 ▸), of up to 1.0697 Å. In contrast, the current data set contains only one crystallographically independent mol­ecule in the asymmetric unit. This mol­ecule overlaps well with one of the two mol­ecules from the previous data set (Fig. 4 ▸*b*, Max·D = 0.2102 Å), but shows poor overlap with the other due to a different orientation of one of the pyridine rings (Fig. 4 ▸*c*, Max·D = 1.1220 Å).

The title compound is used as a means of introducing the 2,2′:6′,2′′-terpyridine moiety in larger architectures (Trigo-López *et al.*, 2016 ▸; Constable *et al.*, 2014 ▸; Lainé *et al.*, 2002 ▸; Perales *et al.*, 2020 ▸; Dong *et al.*, 2019 ▸). This moiety is known for its ability to coordinate metals and is featured in molecules that serve as ligands, polymers and catalysts (Schubert *et al.* 2011*a* ▸,*b* ▸; Winter & Schubert, 2020 ▸; Kainat *et al.*, 2024 ▸).

## Synthesis and crystallization

4-(2,2′:6′,2′′-Terpyridin-4′-yl)aniline was purchased from Sigma Aldrich (CAS:178265–65-1) C_21_H_16_N_4_ and used as received. Dark-brown crystals were obtained upon the evaporation of a concentrated solution of the compound in an aceto­nitrile/methanol solvent system at 298 K. These crystals were characterized by single-crystal X-ray diffraction.

## Refinement

Crystal data, data collection and structure refinement details are summarized in Table 2 ▸.

## Supplementary Material

Crystal structure: contains datablock(s) I. DOI: 10.1107/S2414314626001069/bv4057sup1.cif

Structure factors: contains datablock(s) I. DOI: 10.1107/S2414314626001069/bv4057Isup3.hkl

Supporting information file. DOI: 10.1107/S2414314626001069/bv4057Isup3.cml

CCDC reference: 2527593

Additional supporting information:  crystallographic information; 3D view; checkCIF report

## Figures and Tables

**Figure 3:**
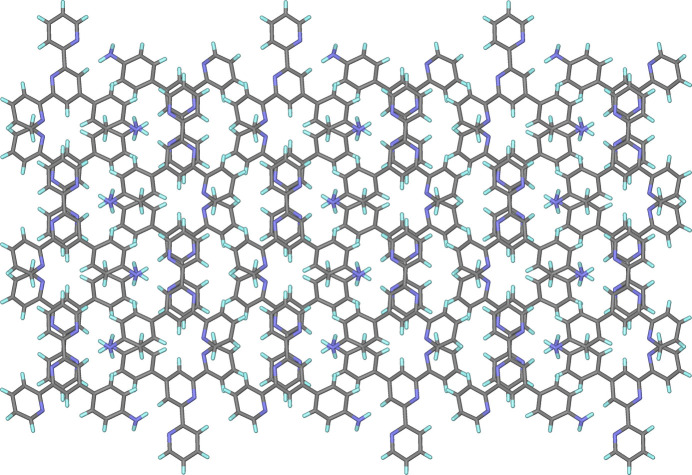
The three-dimensional supra­molecular architecture formed through inter­molecular N—H⋯N and π–π inter­actions.

**Table 1:** Hydrogen-bond geometry (Å, °)

*D*—H⋯*A*	*D*—H	H⋯*A*	*D*⋯*A*	*D*—H⋯*A*
N4—H4*B*⋯N3^i^	0.96 (3)	2.72 (3)	3.154 (3)	108.4 (19)

Symmetry code: (i) 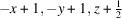.

**Table 2:** Experimental details

Crystal data	
Chemical formula	C_21_H_16_N_4_
*M*_r_	324.38
Crystal system, space group	Orthorhombic, *Pca*2_1_
Temperature (K)	100
*a*, *b*, *c* (Å)	11.3105 (4), 17.3565 (5), 7.9110 (2)
*V* (Å^3^)	1553.01 (8)
*Z*	4
Radiation type	Cu *K*α
μ (mm^−1^)	0.67
Crystal size (mm)	0.14 × 0.12 × 0.10

Data collection	
Diffractometer	Bruker D8 goniometer with Photon III-C14 area detector
Absorption correction	Multi-scan (*SADABS*; Krause *et al.*, 2015 ▸).
*T*_min_, *T*_max_	0.735, 0.864
No. of measured, independent and observed [*I* > 2σ(*I*)] reflections	46277, 2730, 2660
*R*_int_	0.043
(sin θ/λ)_max_ (Å^−1^)	0.596

Refinement	
*R*[*F*^2^ > 2σ(*F*^2^)], *wR*(*F*^2^), *S*	0.028, 0.077, 1.09
No. of reflections	2730
No. of parameters	234
No. of restraints	1
H-atom treatment	H atoms treated by a mixture of independent and constrained refinement
Δρ_max_, Δρ_min_ (e Å^−3^)	0.14, −0.15
Absolute structure	Flack *x* determined using 1188 quotients [(*I*^+^)−(*I*^−^)]/[(*I*^+^)+(*I*^−^)] (Parsons *et al.*, 2013 ▸)
Absolute structure parameter	0.0 (2)

Computer programs: *APEX5* (Bruker, 2023 ▸), *SAINT* (Bruker, 2019 ▸), *SHELXT2019* (Sheldrick, 2015*a* ▸), *SHELXL2019* (Sheldrick, 2015*b* ▸) and *SHELXTL* (Sheldrick, 2008 ▸).

## full crystallographic data

### 4-(2,2':6',2''-Terpyridin-4'-yl)aniline Crystal data

**Table:**

C_21_H_16_N_4_	*D*_x_ = 1.387 Mg m^−^^3^
*M**_r_* = 324.38	Cu *K*α radiation, λ = 1.54178 Å
Orthorhombic, *P**c**a*2_1_	Cell parameters from 9698 reflections
*a* = 11.3105 (4) Å	θ = 4.7–66.5°
*b* = 17.3565 (5) Å	µ = 0.67 mm^−^^1^
*c* = 7.9110 (2) Å	*T* = 100 K
*V* = 1553.01 (8) Å^3^	Plate, colorless
*Z* = 4	0.14 × 0.12 × 0.10 mm
*F*(000) = 680	

### 4-(2,2':6',2''-Terpyridin-4'-yl)aniline Data collection

**Table:**

Bruker D8 goniometer with Photon III-C14 area detector diffractometer	2660 reflections with *I* > 2σ(*I*)
Radiation source: IµS microfocus tube	*R*_int_ = 0.043
ω and phi scans	θ_max_ = 66.7°, θ_min_ = 2.6°
Absorption correction: multi-scan (SADABS; Krause *et al.*, 2015).	*h* = −11→13
*T*_min_ = 0.735, *T*_max_ = 0.864	*k* = −20→20
46277 measured reflections	*l* = −9→9
2730 independent reflections	

### 4-(2,2':6',2''-Terpyridin-4'-yl)aniline Refinement

**Table:**

Refinement on *F*^2^	Hydrogen site location: mixed
Least-squares matrix: full	H atoms treated by a mixture of independent and constrained refinement
*R*[*F*^2^ > 2σ(*F*^2^)] = 0.028	*w* = 1/[σ^2^(*F*_o_^2^) + (0.0442*P*)^2^ + 0.2595*P*] where *P* = (*F*_o_^2^ + 2*F*_c_^2^)/3
*wR*(*F*^2^) = 0.077	(Δ/σ)_max_ < 0.001
*S* = 1.09	Δρ_max_ = 0.14 e Å^−^^3^
2730 reflections	Δρ_min_ = −0.15 e Å^−^^3^
234 parameters	Absolute structure: Flack *x* determined using 1188 quotients [(*I*^+^)-(*I*^-^)]/[(*I*^+^)+(*I*^-^)] (Parsons *et al.*, 2013)
1 restraint	Absolute structure parameter: 0.0 (2)
Primary atom site location: dual	

### 4-(2,2':6',2''-Terpyridin-4'-yl)aniline Special details

**Table:**

Geometry. All esds (except the esd in the dihedral angle between two l.s. planes) are estimated using the full covariance matrix. The cell esds are taken into account individually in the estimation of esds in distances, angles and torsion angles; correlations between esds in cell parameters are only used when they are defined by crystal symmetry. An approximate (isotropic) treatment of cell esds is used for estimating esds involving l.s. planes.
Refinement. Refinement of F^2^ against ALL reflections. The weighted R-factor wR and goodness of fit S are based on F^2^, conventional R-factors R are based on F, with F set to zero for negative F^2^. The threshold expression of F^2^ > 2sigma(F^2^) is used only for calculating R-factors(gt) etc. and is not relevant to the choice of reflections for refinement. R-factors based on F^2^ are statistically about twice as large as those based on F, and R- factors based on all data will be even larger. All non-H atoms were located in difference-Fourier maps, and then refined anisotropically. . Analysis of the absolute structure using likelihood methods (Hooft, *et al.*, 2008) was performed using PLATON (Spek, 2009). The results also indicated that the absolute structure had been correctly assigned. The method calculated that the probability that the structure is inverted is 0.2E-10.The carbon-bound H atoms were placed in calculated positions and refined isotropically using the riding model, with C—H distances ranging from 0.95 Å and *U*_iso_(H) set to 1.2 *U*_eq_(C). The nitrogen-bound H atoms were were located in difference-Fourier maps, and then refined freely.

### 4-(2,2':6',2''-Terpyridin-4'-yl)aniline Fractional atomic coordinates and isotropic or equivalent isotropic displacement parameters (Å^2^)

**Table:**

	*x*	*y*	*z*	*U*_iso_*/*U*_eq_	
N1	0.69467 (15)	0.86075 (10)	0.3804 (2)	0.0272 (4)	
N2	0.39690 (14)	0.82007 (9)	0.5146 (2)	0.0240 (4)	
N3	0.14473 (15)	0.70669 (10)	0.6424 (2)	0.0301 (4)	
N4	0.75980 (19)	0.44154 (11)	0.9566 (3)	0.0364 (5)	
H4A	0.822 (3)	0.4522 (15)	1.026 (4)	0.048 (8)*	
H4B	0.715 (2)	0.3956 (17)	0.976 (4)	0.052 (8)*	
C1	0.7536 (2)	0.91333 (12)	0.2907 (3)	0.0299 (5)	
H1	0.834151	0.903192	0.264762	0.036*	
C2	0.70507 (19)	0.98175 (13)	0.2328 (3)	0.0314 (5)	
H2	0.750727	1.017411	0.169298	0.038*	
C3	0.5876 (2)	0.99629 (13)	0.2710 (3)	0.0317 (5)	
H3	0.551174	1.042731	0.234521	0.038*	
C4	0.52393 (19)	0.94264 (12)	0.3625 (3)	0.0278 (5)	
H4	0.443089	0.951447	0.388699	0.033*	
C5	0.58030 (17)	0.87523 (11)	0.4158 (3)	0.0242 (4)	
C6	0.51511 (17)	0.81394 (11)	0.5090 (3)	0.0235 (4)	
C7	0.57655 (15)	0.75270 (12)	0.5821 (3)	0.0245 (4)	
H7	0.660335	0.750702	0.574948	0.029*	
C8	0.51507 (17)	0.69450 (11)	0.6657 (3)	0.0234 (4)	
C9	0.39189 (17)	0.70053 (11)	0.6684 (3)	0.0238 (4)	
H9	0.345844	0.662200	0.723321	0.029*	
C10	0.33687 (16)	0.76277 (11)	0.5906 (3)	0.0237 (4)	
C11	0.20508 (16)	0.76825 (11)	0.5855 (3)	0.0248 (4)	
C12	0.14940 (17)	0.83300 (12)	0.5207 (3)	0.0282 (4)	
H12	0.194414	0.875495	0.480903	0.034*	
C13	0.02703 (18)	0.83466 (12)	0.5151 (3)	0.0311 (5)	
H13	−0.013144	0.878785	0.473220	0.037*	
C14	−0.03546 (17)	0.77184 (12)	0.5706 (3)	0.0308 (5)	
H14	−0.119402	0.771222	0.566808	0.037*	
C15	0.02704 (18)	0.70949 (13)	0.6322 (3)	0.0321 (5)	
H15	−0.016465	0.665894	0.669645	0.039*	
C16	0.57716 (17)	0.62892 (11)	0.7449 (3)	0.0242 (4)	
C17	0.69150 (17)	0.63765 (12)	0.8096 (3)	0.0257 (4)	
H17	0.728850	0.686605	0.803817	0.031*	
C18	0.7511 (2)	0.57657 (12)	0.8817 (3)	0.0277 (4)	
H18	0.828548	0.584060	0.925107	0.033*	
C19	0.69869 (18)	0.50378 (12)	0.8915 (3)	0.0282 (4)	
C20	0.58456 (18)	0.49475 (11)	0.8264 (3)	0.0293 (5)	
H20	0.547567	0.445650	0.830645	0.035*	
C21	0.52508 (17)	0.55630 (11)	0.7561 (3)	0.0264 (4)	
H21	0.447071	0.549060	0.714568	0.032*	

### 4-(2,2':6',2''-Terpyridin-4'-yl)aniline Atomic displacement parameters (Å^2^)

**Table:**

	*U*^11^	*U*^22^	*U*^33^	*U*^12^	*U*^13^	*U*^23^
N1	0.0226 (8)	0.0295 (9)	0.0293 (9)	−0.0012 (7)	−0.0001 (7)	−0.0010 (7)
N2	0.0207 (8)	0.0242 (8)	0.0272 (9)	0.0013 (6)	−0.0004 (7)	−0.0043 (7)
N3	0.0220 (9)	0.0302 (9)	0.0381 (10)	0.0005 (7)	0.0006 (8)	−0.0025 (8)
N4	0.0314 (10)	0.0337 (10)	0.0441 (12)	0.0050 (9)	−0.0015 (9)	0.0077 (9)
C1	0.0229 (10)	0.0368 (11)	0.0301 (12)	−0.0040 (9)	0.0009 (9)	−0.0005 (9)
C2	0.0333 (11)	0.0323 (11)	0.0286 (12)	−0.0071 (8)	0.0015 (9)	0.0026 (10)
C3	0.0357 (12)	0.0276 (10)	0.0317 (12)	0.0004 (8)	−0.0013 (9)	0.0014 (9)
C4	0.0252 (10)	0.0279 (10)	0.0302 (12)	−0.0003 (8)	−0.0001 (8)	−0.0017 (9)
C5	0.0227 (10)	0.0261 (10)	0.0237 (11)	−0.0014 (7)	−0.0015 (8)	−0.0044 (8)
C6	0.0216 (10)	0.0243 (9)	0.0245 (10)	0.0003 (7)	−0.0009 (9)	−0.0049 (8)
C7	0.0182 (8)	0.0266 (9)	0.0287 (10)	−0.0003 (8)	−0.0010 (9)	−0.0035 (8)
C8	0.0214 (10)	0.0238 (10)	0.0250 (10)	0.0005 (7)	−0.0014 (8)	−0.0061 (8)
C9	0.0208 (10)	0.0241 (10)	0.0266 (10)	−0.0023 (7)	0.0007 (8)	−0.0032 (8)
C10	0.0214 (9)	0.0240 (10)	0.0257 (10)	0.0007 (7)	−0.0007 (8)	−0.0060 (8)
C11	0.0208 (9)	0.0271 (10)	0.0264 (10)	0.0006 (7)	−0.0008 (8)	−0.0071 (8)
C12	0.0243 (10)	0.0290 (11)	0.0313 (11)	0.0019 (8)	−0.0014 (9)	−0.0031 (9)
C13	0.0255 (10)	0.0344 (11)	0.0334 (11)	0.0073 (8)	−0.0044 (9)	−0.0041 (10)
C14	0.0196 (10)	0.0388 (12)	0.0338 (12)	0.0023 (8)	−0.0012 (9)	−0.0106 (9)
C15	0.0223 (10)	0.0340 (12)	0.0401 (13)	−0.0024 (8)	0.0010 (9)	−0.0031 (10)
C16	0.0214 (10)	0.0251 (10)	0.0260 (10)	0.0017 (7)	0.0015 (8)	−0.0016 (8)
C17	0.0236 (11)	0.0248 (10)	0.0286 (11)	−0.0008 (7)	0.0004 (9)	−0.0033 (8)
C18	0.0216 (9)	0.0328 (10)	0.0286 (11)	0.0025 (8)	−0.0025 (8)	−0.0024 (9)
C19	0.0272 (10)	0.0301 (11)	0.0272 (11)	0.0049 (8)	0.0035 (9)	0.0016 (9)
C20	0.0261 (11)	0.0250 (10)	0.0367 (12)	−0.0017 (7)	0.0030 (9)	0.0016 (9)
C21	0.0197 (9)	0.0281 (10)	0.0315 (11)	0.0000 (7)	−0.0001 (8)	−0.0027 (8)

### 4-(2,2':6',2''-Terpyridin-4'-yl)aniline Geometric parameters (Å, º)

**Table:**

N1—C1	1.335 (3)	C8—C16	1.477 (3)
N1—C5	1.347 (3)	C9—C10	1.391 (3)
N2—C6	1.342 (2)	C9—H9	0.9500
N2—C10	1.346 (3)	C10—C11	1.494 (2)
N3—C15	1.334 (3)	C11—C12	1.386 (3)
N3—C11	1.346 (3)	C12—C13	1.385 (3)
N4—C19	1.382 (3)	C12—H12	0.9500
N4—H4A	0.91 (3)	C13—C14	1.372 (3)
N4—H4B	0.96 (3)	C13—H13	0.9500
C1—C2	1.386 (3)	C14—C15	1.381 (3)
C1—H1	0.9500	C14—H14	0.9500
C2—C3	1.386 (3)	C15—H15	0.9500
C2—H2	0.9500	C16—C21	1.394 (3)
C3—C4	1.382 (3)	C16—C17	1.399 (3)
C3—H3	0.9500	C17—C18	1.380 (3)
C4—C5	1.398 (3)	C17—H17	0.9500
C4—H4	0.9500	C18—C19	1.398 (3)
C5—C6	1.490 (3)	C18—H18	0.9500
C6—C7	1.396 (3)	C19—C20	1.399 (3)
C7—C8	1.393 (3)	C20—C21	1.380 (3)
C7—H7	0.9500	C20—H20	0.9500
C8—C9	1.397 (3)	C21—H21	0.9500
			
C1—N1—C5	117.55 (18)	C9—C10—C11	120.52 (17)
C6—N2—C10	117.31 (17)	N3—C11—C12	122.48 (17)
C15—N3—C11	117.18 (17)	N3—C11—C10	116.52 (17)
C19—N4—H4A	116.8 (17)	C12—C11—C10	120.98 (18)
C19—N4—H4B	116.7 (17)	C13—C12—C11	118.87 (19)
H4A—N4—H4B	119 (3)	C13—C12—H12	120.6
N1—C1—C2	124.3 (2)	C11—C12—H12	120.6
N1—C1—H1	117.9	C14—C13—C12	119.20 (19)
C2—C1—H1	117.9	C14—C13—H13	120.4
C3—C2—C1	117.7 (2)	C12—C13—H13	120.4
C3—C2—H2	121.2	C13—C14—C15	118.17 (18)
C1—C2—H2	121.2	C13—C14—H14	120.9
C4—C3—C2	119.4 (2)	C15—C14—H14	120.9
C4—C3—H3	120.3	N3—C15—C14	124.1 (2)
C2—C3—H3	120.3	N3—C15—H15	118.0
C3—C4—C5	119.0 (2)	C14—C15—H15	118.0
C3—C4—H4	120.5	C21—C16—C17	117.72 (18)
C5—C4—H4	120.5	C21—C16—C8	121.52 (18)
N1—C5—C4	122.12 (19)	C17—C16—C8	120.75 (18)
N1—C5—C6	116.40 (17)	C18—C17—C16	121.30 (19)
C4—C5—C6	121.43 (18)	C18—C17—H17	119.3
N2—C6—C7	122.87 (18)	C16—C17—H17	119.3
N2—C6—C5	116.93 (17)	C17—C18—C19	120.7 (2)
C7—C6—C5	120.18 (17)	C17—C18—H18	119.7
C8—C7—C6	120.03 (16)	C19—C18—H18	119.7
C8—C7—H7	120.0	N4—C19—C18	121.0 (2)
C6—C7—H7	120.0	N4—C19—C20	120.7 (2)
C7—C8—C9	116.78 (18)	C18—C19—C20	118.18 (19)
C7—C8—C16	121.52 (18)	C21—C20—C19	120.78 (19)
C9—C8—C16	121.69 (18)	C21—C20—H20	119.6
C10—C9—C8	119.84 (18)	C19—C20—H20	119.6
C10—C9—H9	120.1	C20—C21—C16	121.32 (19)
C8—C9—H9	120.1	C20—C21—H21	119.3
N2—C10—C9	123.10 (17)	C16—C21—H21	119.3
N2—C10—C11	116.37 (17)		
			
C5—N1—C1—C2	0.4 (3)	C15—N3—C11—C10	−177.32 (18)
N1—C1—C2—C3	0.0 (3)	N2—C10—C11—N3	172.02 (19)
C1—C2—C3—C4	−0.6 (3)	C9—C10—C11—N3	−7.1 (3)
C2—C3—C4—C5	0.6 (3)	N2—C10—C11—C12	−6.3 (3)
C1—N1—C5—C4	−0.3 (3)	C9—C10—C11—C12	174.62 (19)
C1—N1—C5—C6	177.18 (18)	N3—C11—C12—C13	0.3 (3)
C3—C4—C5—N1	−0.2 (3)	C10—C11—C12—C13	178.51 (18)
C3—C4—C5—C6	−177.60 (19)	C11—C12—C13—C14	−1.2 (3)
C10—N2—C6—C7	−2.0 (3)	C12—C13—C14—C15	0.8 (3)
C10—N2—C6—C5	176.47 (17)	C11—N3—C15—C14	−1.4 (3)
N1—C5—C6—N2	−166.66 (18)	C13—C14—C15—N3	0.5 (4)
C4—C5—C6—N2	10.9 (3)	C7—C8—C16—C21	−149.1 (2)
N1—C5—C6—C7	11.8 (3)	C9—C8—C16—C21	29.8 (3)
C4—C5—C6—C7	−170.62 (19)	C7—C8—C16—C17	30.1 (3)
N2—C6—C7—C8	−0.1 (3)	C9—C8—C16—C17	−150.9 (2)
C5—C6—C7—C8	−178.45 (19)	C21—C16—C17—C18	0.2 (3)
C6—C7—C8—C9	1.2 (3)	C8—C16—C17—C18	−179.0 (2)
C6—C7—C8—C16	−179.86 (19)	C16—C17—C18—C19	0.2 (3)
C7—C8—C9—C10	−0.3 (3)	C17—C18—C19—N4	177.0 (2)
C16—C8—C9—C10	−179.25 (19)	C17—C18—C19—C20	0.0 (3)
C6—N2—C10—C9	2.9 (3)	N4—C19—C20—C21	−177.7 (2)
C6—N2—C10—C11	−176.15 (17)	C18—C19—C20—C21	−0.7 (3)
C8—C9—C10—N2	−1.8 (3)	C19—C20—C21—C16	1.1 (3)
C8—C9—C10—C11	177.20 (17)	C17—C16—C21—C20	−0.9 (3)
C15—N3—C11—C12	0.9 (3)	C8—C16—C21—C20	178.3 (2)

### 4-(2,2':6',2''-Terpyridin-4'-yl)aniline Hydrogen-bond geometry (Å, º)

**Table:**

*D*—H···*A*	*D*—H	H···*A*	*D*···*A*	*D*—H···*A*
N4—H4*B*···N3^i^	0.96 (3)	2.72 (3)	3.154 (3)	108.4 (19)

Symmetry code: (i) −*x*+1, −*y*+1, *z*+1/2.
